# Climate Drives Modeled Forest Carbon Cycling Resistance and Resilience in the Upper Great Lakes Region, USA

**DOI:** 10.1029/2021JG006587

**Published:** 2022-01-13

**Authors:** Kalyn Dorheim, Christopher M. Gough, Lisa T. Haber, Kayla C. Mathes, Alexey N. Shiklomanov, Ben Bond‐Lamberty

**Affiliations:** ^1^ Joint Global Change Research Institute Pacific Northwest National Laboratory College Park MD USA; ^2^ Department of Biology Virginia Commonwealth University Richmond VA USA; ^3^ NASA Goddard Space Flight Center Greenbelt MD USA

**Keywords:** carbon cycle

## Abstract

Forests dominate the global terrestrial carbon budget, but their ability to continue doing so in the face of a changing climate is uncertain. A key uncertainty is how forests will respond to (resistance) and recover from (resilience) rising levels of disturbance of varying intensities. This knowledge gap can optimally be addressed by integrating manipulative field experiments with ecophysiological modeling. We used the Ecosystem Demography‐2.2 (ED‐2.2) model to project carbon fluxes for a northern temperate deciduous forest subjected to a real‐world disturbance severity manipulation experiment. ED‐2.2 was run for 150 years, starting from near bare ground in 1900 (approximating the clear‐cut conditions at the time), and subjected to three disturbance treatments under an ensemble of climate conditions. Both disturbance severity and climate strongly affected carbon fluxes such as gross primary production (GPP), and interacted with one another. We then calculated resistance and resilience, two dimensions of ecosystem stability. Modeled GPP exhibited a two‐fold decrease in mean resistance across disturbance severities of 45%, 65%, and 85% mortality; conversely, resilience increased by a factor of two with increasing disturbance severity. This pattern held for net primary production and net ecosystem production, indicating a trade‐off in which greater initial declines were followed by faster recovery. Notably, however, heterotrophic respiration responded more slowly to disturbance, and it's highly variable response was affected by different drivers. This work provides insight into how future conditions might affect the functional stability of mature forests in this region under ongoing climate change and changing disturbance regimes.

## Key Points

- Climatic conditions affect how forests will respond to and recover from disturbances
- Disturbance severity strongly affects carbon fluxes

## 1 Introduction

Historically forests have acted as a large terrestrial carbon (C) sink (Pan et al., 2011; Tagesson et al., 2020), but their future role in the global C budget is uncertain. From 2010 to 2019 the terrestrial land sink was an estimated 12.5 Gt carbon dioxide (CO_2_) per year (Friedlingstein et al., 2020) with the majority of this stored in forested ecosystems (Tagesson et al., 2020). However, this C sequestration capability is sensitive to a number of natural and anthropogenic influences such as CO_2_ enrichment, drought, wildfires, pests, windfall events, land management, and change in land use and land cover (Davidson et al., 2012; Schimel et al., 2015; Yang et al., 2018). Ultimately the future role and efficacy of forests in the C cycle remains uncertain, especially with convergence of rapidly changing climate and disturbance.

Disturbances constitute a particularly large uncertainty for forest C dynamics (McKinley et al., 2011). For example, a single storm event in 2005 was responsible for moving 0.09–0.11 Pg C from live to dead biomass pools (Chambers et al., 2007). At the same time, fires within the U.S. release about 0.06 petagrams of C per year (Wiedinmyer & Neff, 2007), and 1.8–3.0 Pg C yr^−1^ globally (van der Werf et al., 2017). Disturbance events such as storms and fires exhibit high interannual variability and have a broad range of biogeochemical and ecological effects. Many studies have investigated water, nutrients, and C cycling in post‐disturbance ecosystems (Amiro et al., 2010; Edburg et al., 2012; Gough et al., 2013; Matheny et al., 2014). However, the effects of interacting disturbances (Liang et al., 2016), disturbance severity (Stuart‐Haëntjens et al., 2015), disturbance‐climate interactions, and the longer‐term consequences of disturbances are difficult to study through observations and, therefore, less well understood (Gough, 2021).

Manipulative field studies and ecological models offer complementary insights and means to investigate post‐disturbance C cycling stability. Large scale field experiments where mortality was manipulated include the Forest Accelerated Succession Experiment (FASET) (Gough et al., 2013; Gough, Bohrer & Curtis, 2021; Gough, Bohrer, Hardiman, et al., 2021) and the Forest Resilience Threshold Experiment (FoRTE), both conducted at the University of Michigan Biological Station (UMBS). While these studies have resulted in a number of advances (Curtis & Gough, 2018; Frasson et al., 2015), it has taken FASET over a decade to generate the results needed to test the experiment's core hypotheses (Gough, Bohrer & Curtis, 2021; Gough, Bohrer, Hardiman, et al., 2021). In contrast, ecosystem models which can be either take a process, statistical, or some mixed approach, offer the ability to look at longer time horizons; produce a large number of replicates; may provide data at more granular time intervals than would be feasible through field sampling; vary parameters and examine additional treatment effects not easily manipulated in the field (long‐term climate); and make falsifiable predictions (Dietze, 2017). For example, ecosystem models can be used to quickly predict the decade‐long effects of different disturbance sources (Dietze & Matthes, 2014). Combining a manipulative field experiment and ecosystem modeling leverages the advantages of both approaches, helping to inform model structure, develop field testable hypotheses, and prioritized field data collection (Dietze et al., 2013; Medlyn et al., 2015; Keenan et al., 2013). For this reason several recent syntheses have called for replicated, manipulative experiments closely linked with modeling analyses to investigate forest responses to disturbance (Amiro et al., 2010; Goetz et al., 2012; Norby et al., 2015; Medlyn et al., 2015).

**Figure 1:**
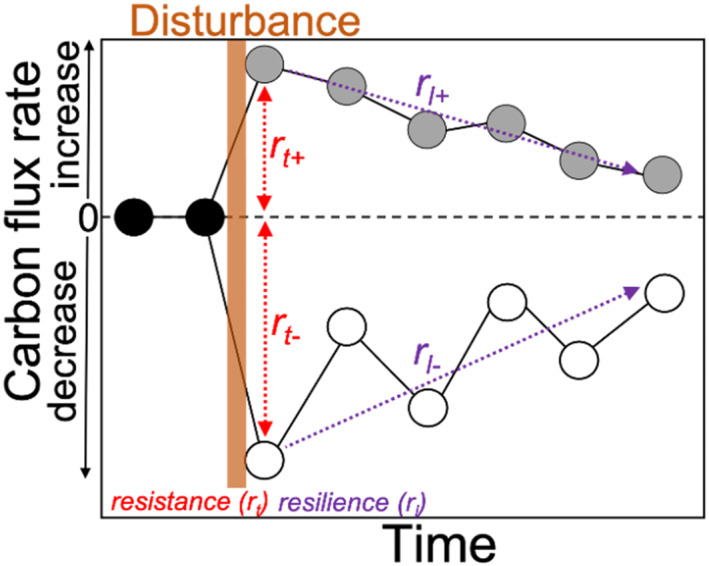
Conceptual diagram of the multi‐dimensional stability framework. We focus on two metrics of stability: *resistance* and *resilience* (see Methods) adopted from Mathes et al. (2021).

Multi‐dimensional stability frameworks provide a basis for systematically decomposing and analyzing various features of disturbance response facilitating comparisons between different sources (i.e., studies, field or model observations) (Egli et al., 2019; Harrison, 1979; Mathes et al., 2021; Pimm, 1984). As part of FoRTE (Grigri et al., 2020; Gough, Bohrer & Curtis, 2021; Gough, Bohrer, Hardiman, et al., 2021), we have adopted the Hillebrand et al. (2018) multidimensional stability framework, described by Mathes et al. (2021), to quantify different components of ecosystem stability and disturbance response. In this manuscript, we focus on two dimensions of stability, *resistance* and *resilience* (Figure 1), in which *resistance* captures the maximum change in magnitude and direction following disturbance, and *resistance* captures the rate of change as the structure and/or function of a system recovers.

The objective of our study was to use an ecosystem model to make and interpret projections of C cycling resilience and resistance in a deciduous forest subjected to varying levels of disturbance severity under different climate scenarios. We used the Ecosystem Demography model, version 2.2 (ED‐2.2) (Longo et al., 2019; Medvigy et al., 2009; Moorcroft et al., 2001), a process based ecosystem model capable of modeling fine‐scale vegetation and C cycle dynamics, for this dual modeling/experimental study. We aim to address the following questions: (a) What do long‐term (multi‐decadal) projections suggest about C cycle stability following different levels of disturbance severity and under different climate scenarios? (b) Is there a trade‐off, as theorized, between resilience and resistance? (c) What is the interacting effect of climate and disturbance severity on C cycling responses to disturbance? (d) How do ED‐2.2 projections of C cycling response compare to what has been observed to date in the ongoing FoRTE experiment? Lastly, what insights can we gain regarding ecology and the priorities for future field measurements and forest management?

## 2 Materials and Methods

The large‐scale manipulative field component of FoRTE was conducted at the University of Michigan Biological Station (UMBS, 45.56°N, 84.67°W). Located in the northern lower peninsula of Michigan, USA, the region receives an average of 817 mm of precipitation a year with an average annual temperature of 5.5°C (Gough et al., 2013) and is classified as a cold temperate forest. At the beginning of the twentieth century, the site—like most of the region—was clear‐cut harvested, and the regrown century‐old deciduous forest now consists of declining bigtooth, trembling aspen, and paper birch (*Populus grandidentata*, *P. tremuloides*, and *Betula papyrifera*, respectively) and increasing later successional northern red oak, red maple, and white pine (Atkins et al., 2020). UMBS soils are sandy, excessively drained, mixed frigid Entic Haplorthods with little relief (Hardiman et al., 2011). Due to the site's regionally representative disturbance history, many disturbance‐ and succession‐focused field studies have been conducted at UMBS (Gough et al., 2013, Gough, Bohrer & Curtis, 2021; Gough, Bohrer, Hardiman, et al., 2021; Stuart‐Haëntjens et al., 2015).

In May 2019, prior to leaf‐out, the FoRTE treatments were applied to circular 0.5 ha plots at UMBS. Hereafter, the disturbance treatment severity (0, 45, 65, and 85) refers to the targeted percent of gross leaf area loss per plot achieved via stem girdling. Stem girdling at our site leads to tree mortality after trees’ carbohydrate reserves are exhausted, typically within 2–3 years (Gough et al., 2013). Each severity treatment was applied within four different land ecosystems, representing a range in site productivity characteristic of the forests of the Upper Great Lakes Region (Atkins et al., 2020). Starting a year before the disturbance treatments were applied in 2018 C and nitrogen cycling, canopy structure, photosynthesis, biomass growth, and soil respiration were recorded (Grigri et al., 2020), documented, published, and distributed via the *fortedata* R package (Atkins et al., 2020).

### 2.1 Ecological Modeling

The Ecosystem Demography Model version 2.2 (ED‐2.2) was used in the modeling component of FoRTE. ED‐2.2 is a cohort‐based model, meaning that it represents groups of plants similar in size and functional type. This allows ED‐2.2 to represent forest heterogeneity without the computational burden of modeling each tree individually, in turn allowing the model to represent the small‐scale heterogeneity arising from disturbance and tree mortality. Within each cohort ED‐2.2 solves systems of differential equations that describe the biophysical and physiological processes that move energy, water, and C through a terrestrial ecosystem (Longo et al., 2019).The unique capabilities of ED‐2.2, including multiple plant functional types, the multiple disturbance mechanisms implemented by Hurtt et al. (2002) and Albani et al. (2006), along with the ability to run ED‐2.2 offline with prescribed climatology, made it an ideal model for our use.

ED‐2.2 was set up with its default structure configuration, completely shaded crown and no trait plasticity. The model was parameterized with UMBS information such as soil characteristics and profile obtained from site‐level observations (Gough et al., 2010) with soil moisture data from the UMBS Ameriflux ancillary data (https://ameriflux.lbl.gov/sites/siteinfo/US-UMd). The meteorological inputs were derived from historical observations, described in detail in the following section. To best reflect the tree inventories of the FoRTE UMBS study plots, ED‐2.2 was set to model three plant functional groups: early, mid, and late temperate deciduous (“hardwood”) (Gough et al., 2013).

ED‐2.2 implements multiple types of disturbance events, including land clearing, land abandonment, and selective forest harvesting, as well as the ability to prescribe specific disturbance events (Longo et al., 2019). However, ED‐2.2 does not support a specific tree‐girdling disturbance mechanism, and thus the FoRTE treatments were implemented using the model's selective harvest capability. This disturbance type removes the specified fraction (45%, 65%, or 85%) of plant biomass, leading to mortality which varies as a function of the C balance (Albani et al., 2006). This provides a disturbance mechanism that is similar over the short term to girdling: aboveground biomass is removed (via harvest, in the model) or immobilized (via girdling, in the field experiment); in both cases, the photosynthate supply to the roots is disrupted, producing relatively rapid (relative to the period of modeling) tree mortality. We review strengths and limitations of using this approach in the discussion.

### 2.2 Climate Data

**Figure 2:**
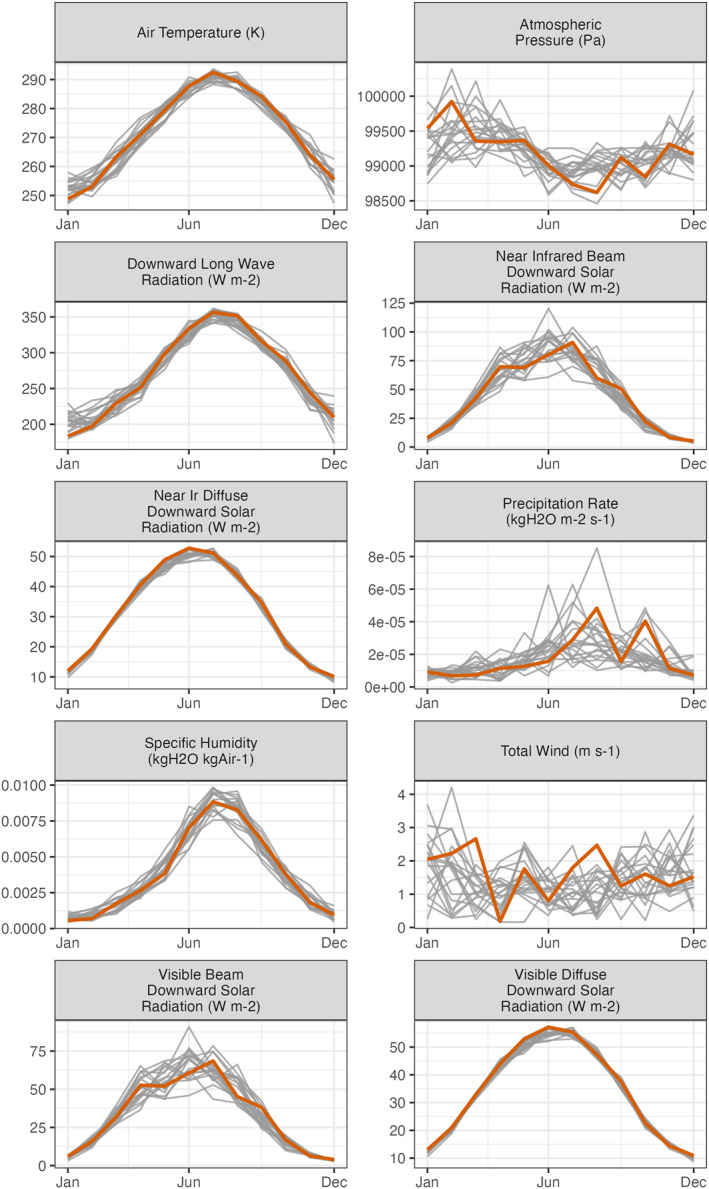
The average monthly meteorological values from the ensemble of idealized climate inputs. Each line represents a different climate scenario constructed from observed meteorological conditions at the study site; the thick orange line highlights observed weather in 2019, the year of the FoRTE experimental disturbance (see Methods). For each climate ensemble member, the sub‐daily values are looped over to generate a complete time series from 1900 to 2050 with a constant annual cycle over the entire run. See (Figure S1 in Supporting Information S1) for the annual mean meteorological values.

Meteorological data were obtained for UMBS from the North American Regional Reanalysis (NARR) (Mesinger et al., 2006).These data included three‐hourly downward longwave radiation, near‐infrared beam downward solar radiation, near‐infrared diffuse downward solar radiation, visible beam downward solar radiation, visible diffuse downward solar radiation, precipitation rate, atmospheric pressure, geopotential height, zonal wind, meridional wind, specific humidity, and air temperature. Climate and weather variability can have significant effects on forest model growth and study conclusions (Horemans et al., 2021); to isolate the effect of climate on C cycling stability across a disturbance severity gradient, we used *idealized climate trajectories* for the ED‐2.2 runs to systematically introduce climate variability while removing year‐to‐year variability within each trajectory. A single year was uniformly selected from the available NARR historical data record and repeated 150 times to create an input data set from 1900 to 2050. This process was repeated 20 times to create an ensemble of runs, each one corresponding to a different climate (Figure 2).

In total ED‐2.2 was run 80 times, starting in 1900 from “near‐bare ground” conditions until 2050. Each of the 20 realizations was run four times, one for each of the FoRTE severity treatments. The FoRTE‐analogous disturbance treatments were applied as a 0, 0.45, 0.65, and 0.85 fractional removal of a portion of C in the above‐ground, foliage, and storage biomass pools using ED‐2.2 selective harvest mechanism. Only the C in the below‐ground biomass remained undisturbed.

### 2.3 Resistance & Resilience

We adopted the Hillebrand et al. (2018) framework to quantify two dimensions of ecosystem stability: *resistance* and resilience (Figure 1). This standardized framework and the normalization of C cycling responses to disturbance lends itself to comparing functional responses to disturbances across sources, allowing us to compare and synthesize ED‐2.2 results with observations from the UMBS. *Resistance* and *resilience* are calculated from the log ratio of the disturbance to control flux; the disturbance flux refers to results from the 45%, 65%, and 85% mortality FoRTE runs, while the control run refers to the 0% control. *Resistance* captures the maximum response in magnitude and direction to the disturbance response. After *resistance,* when the system begins to recover, *resilience* then captures the rate of change as the slope of the log ratio, calculated with linear regression or segmented linear regression.

The ED‐2.2 model has a wide range of outputs at many different temporal, spatial, and demographic scales (Longo et al., 2019). Here we generally analyze four of them: annual gross primary productivity (GPP), net primary productivity (NPP), net ecosystem productivity (NEP), and heterotrophic respiration (Rh) fluxes, which were processed from ED‐2.2 hdf5 files using R 3.6.3. We focused on these because they form the primary basis for understanding ecosystem‐scale C cycling: photosynthesis, plant growth, C balance, and microbial respiration.

To examine how the idealized climate inputs affected resistance and resilience, we first use Principal Coordinate Analysis (Zuur et al., 2007) to identify correlated predictors (i.e., climate variables that were highly correlated with each other). Based on the results of this analysis (Figure S2 in Supporting Information S1) we removed the near infrared beam downward solar radiation and near infrared diffuse downward solar radiation variables, as these were highly correlated with their visible‐light counterparts. The remaining nine meteorological input variables along with the pre‐disturbance productivity (GPP in 2000), were used as potential predictors in a Random Forest model designed to quantify predictor importance. We built a separate model for each stability metric, that is, one for *resistance* and another for *resilience*. The Random Forest algorithm (Breiman, 2001) is a nonparametric machine learning technique for classification and regression; as a data‐driven methodology, it makes no a priori theoretical assumptions about drivers or behavior, and is increasingly used in earth and ecological sciences (Reichstein et al., 2019). The algorithm predicts by aggregating regression trees constructed using different random samples of the data, and choosing splits of the trees from subsets of the available predictors, which are randomly chosen at each node. We used the default settings of the R *randomForest* package (Liaw & Wiener, 2002), in particular *ntree* (number of trees) = 500 and *mtry* (number of candidate variables sampled at each split) = 3.

## 3 Data Availability

R 3.6.3 (R Development Core Team, 2018) was used to process ED‐2.2 hdf5 results, calculate the metrics quantifying the dimensions of stability and perform the statistical analyses. All these materials along with the code used to generate the climate ensemble inputs, harvest treatments, and ED‐2.2 configuration files are available and well documented at https://github.com/forTExperiment/forte-disturbance, archived on zenodo with the DOI: 10.5281/zenodo.5636671 (Dorheim & Bond‐Lamberty, 2021).

## 4 Results

**Figure 3:**
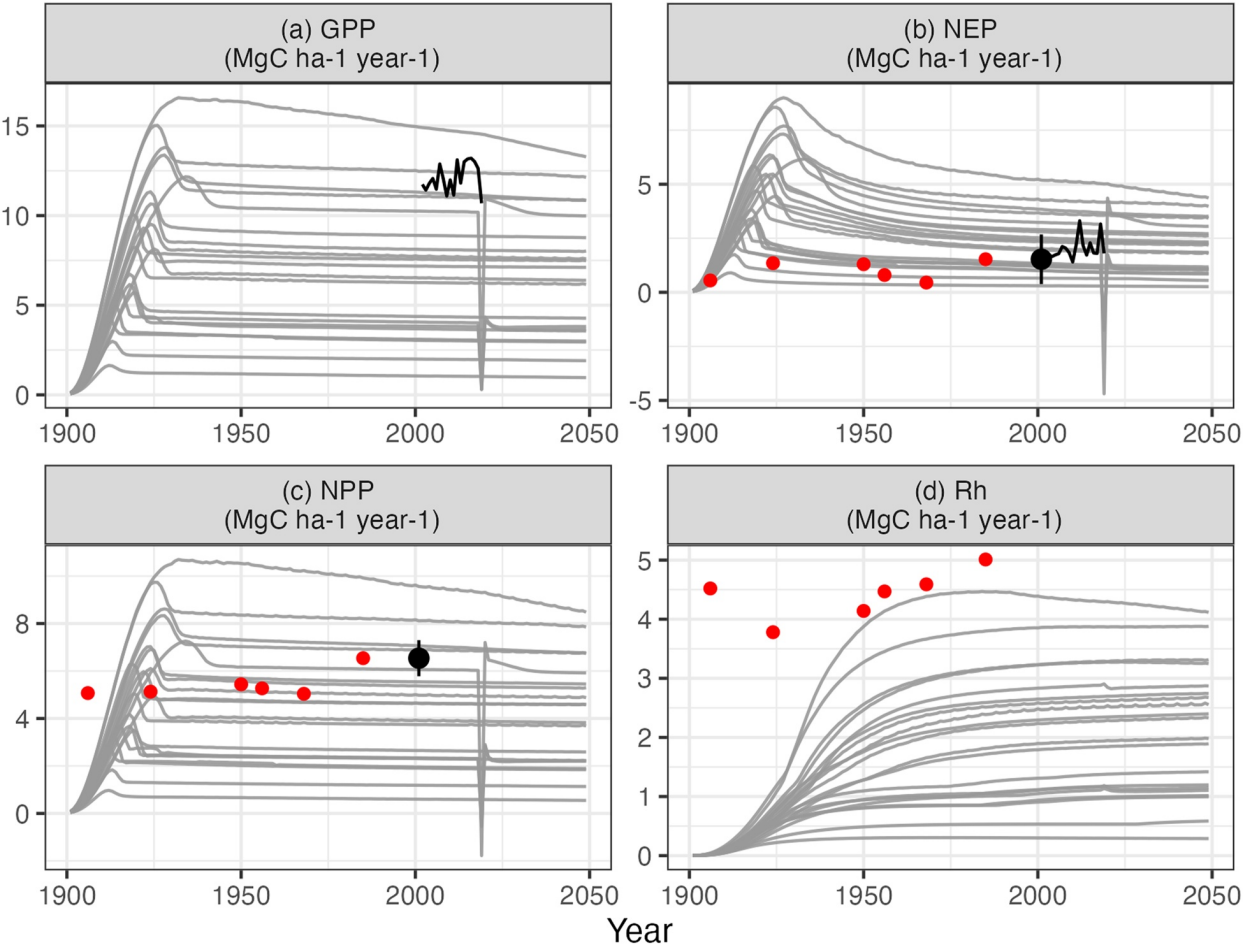
Annual ED‐2.2 output fluxes of gross primary productivity (GPP), net ecosystem productivity (NEP), net primary productivity (NPP), and heterotrophic respiration (Rh) for the 0% disturbance severity runs (control runs). These flux rates were used as the control fluxes when calculating the Hillebrand et al. (2018) dimensions of ecosystem stability (cf. Figure 1). Each of the gray lines is an ED‐2.2 output from the different climate realizations. The black NEP and NPP points refer to the mean observed NEP and NPP UMBS values from Table 2 of Gough et al. (2008) while the black lines correspond to GPP and NEP Ameriflux observations (Gough, Bohrer & Curtis, 2021; Gough, Bohrer, Hardiman, et al., 2021). Red dots are data from a regional chronosequence (Gough et al., 2007) and thus not direct observations of the study site. The dramatic decline and recovery of GPP, NEP, and NPP occurs within a single ensemble member for the control run and all FoRTE disturbance runs. It occurs as the result of a mortality event caused by that set of meteorological inputs.

The control runs (0% severity treatment) produce successional C cycling dynamics that are comparable to those observed at UMBS. The simulated forests were established in 1900 from near bare ground conditions, matching the early twentieth‐century clearcuts that occurred in the real‐world UMBS region, with very small C fluxes as illustrated in Figure 3. Over the course of the century the C fluxes increase as the simulated forest grows and matures. By the late twentieth century the simulated forest C fluxes have reached a stable equilibrium (in part because by design the experiments exclude year‐to‐year weather variability within a single run; see Methods). The ensemble mean C fluxes in 2001 were 6.9 ± 3.8 (ensemble mean ± standard deviation), 2.3 ± 1.3, 4.3 ± 2.4, and 2.1 ± 1.1 megagrams of C per hectare per year (MgC ha^−1^ year^−1^) for GPP, NEP, NPP, and Rh, respectively. The ED ensemble aboveground biomass mean for 2001 was 5.6 kg C m^−2^ (kilogram of C per square meter).

**Figure 4:**
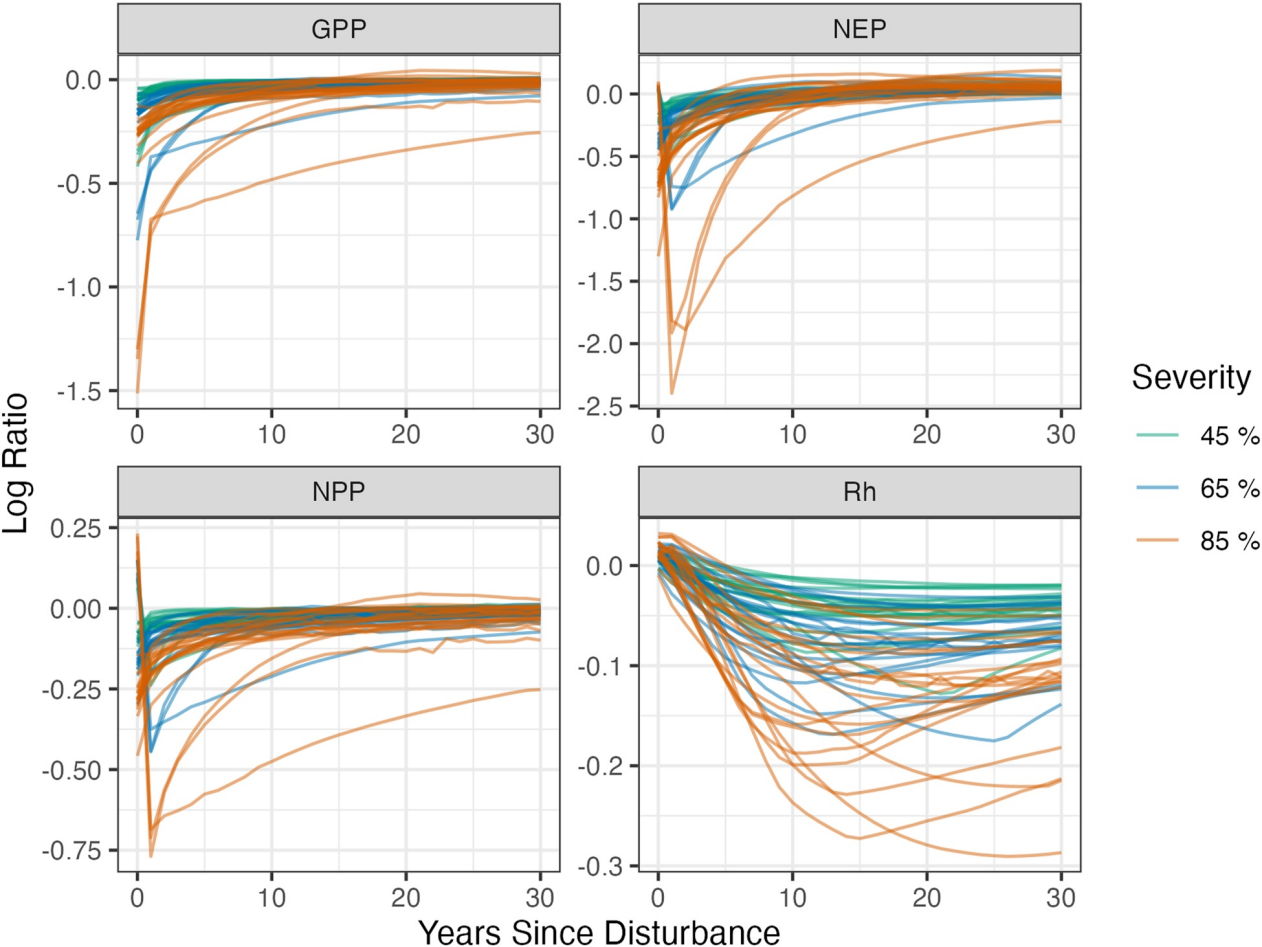
Log ratio of the disturbance to control fluxes for gross primary production (a, GPP), net ecosystem production (b, NEP), net primary production (c, NPP), and heterotrophic respiration (d, Rh) for years since the simulated disturbance. The green, blue, and orange lines refer to the 45%, 65%, and 85% FoRTE disturbance severity treatments respectively. Each panel has 20 individual lines, one for each of the idealized climate scenarios (cf. Figure 2). The log‐ratio time series are used to calculate *resistance* and *resilience*.

In 2019, the model experiences our “FoRTE disturbance,” in which an aboveground harvest is applied in each run at the FoRTE treatment levels of 0% (control), 45%, 65%, or 85% mortality. Across severity treatments, the disturbance event caused the C fluxes to decrease (visualized in Figure 4 as log ratios). Generally larger—more negative—responses were observed with increasing treatment severity; however, these responses were highly dependent on the climate realization (Figure 4). GPP exhibited the most immediate and severe response to disturbance (Figure 4a) while the Rh response was more gradual and peaked later (Figure 4d). In some instances it took Rh up to 20 years to capture the full disturbance response. The log‐ratio time series was most consistent in terms of shape across the ensemble for GPP (Figure 4a) whereas Rh (Figure 4b) varied the most.

**Figure 5:**
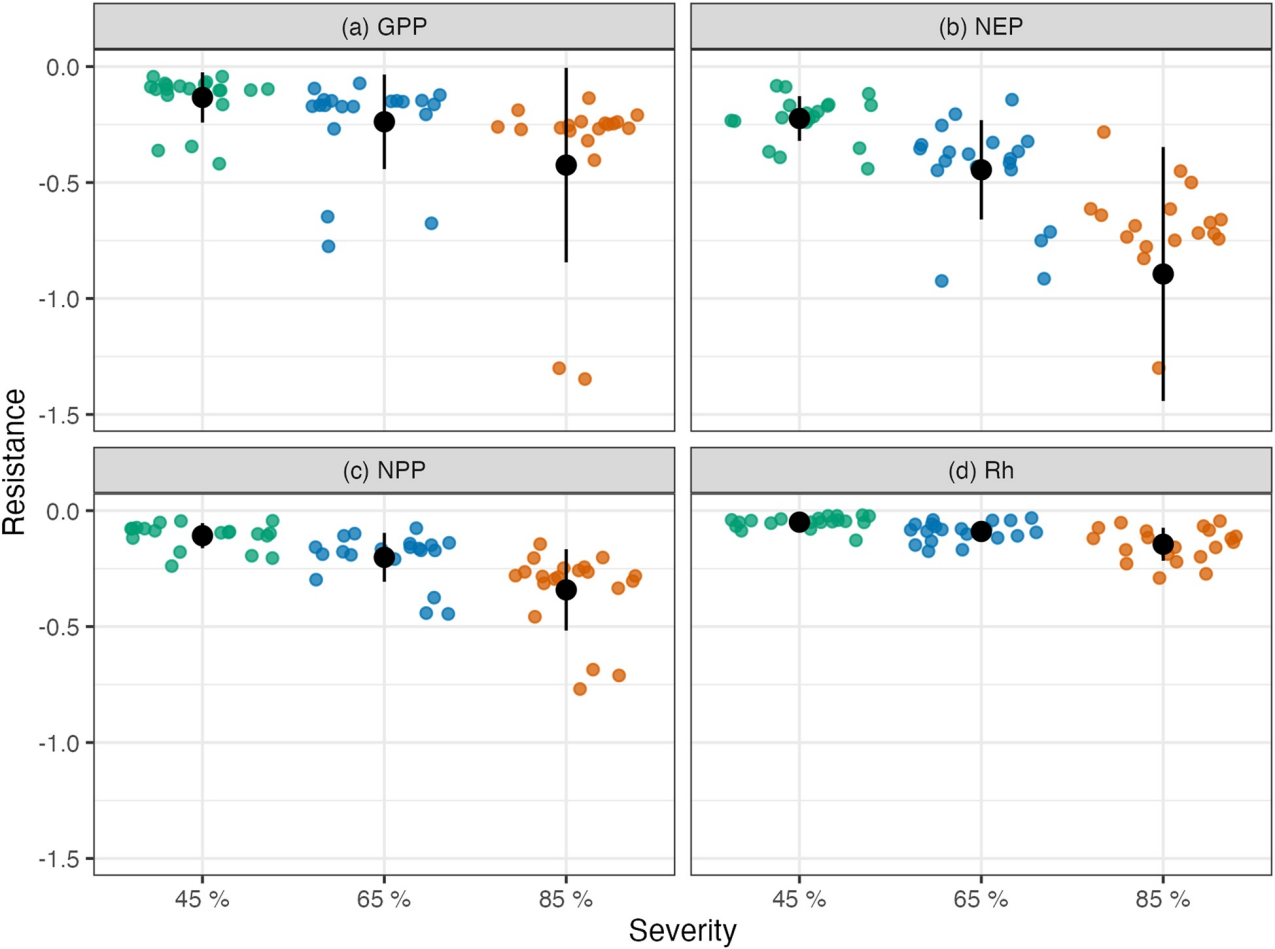
*Resistance*, the largest change in magnitude and direction of log‐ratio of disturbance to control flux for gross primary production (a, GPP), net ecosystem production (b, NEP), net primary production (c, NPP), and heterotrophic respiration (d, Rh). Each of the FoRTE disturbance severity groups has 20 individual points, one for each of the idealized climate scenarios. The black point with error bar represents the mean *resistance* (Table 1) ± the standard deviation for each variable and treatment group. For clarity four *resistance* values less than −1.5 are not shown here, but contributed to the mean and standard deviation.

**Figure 6:**
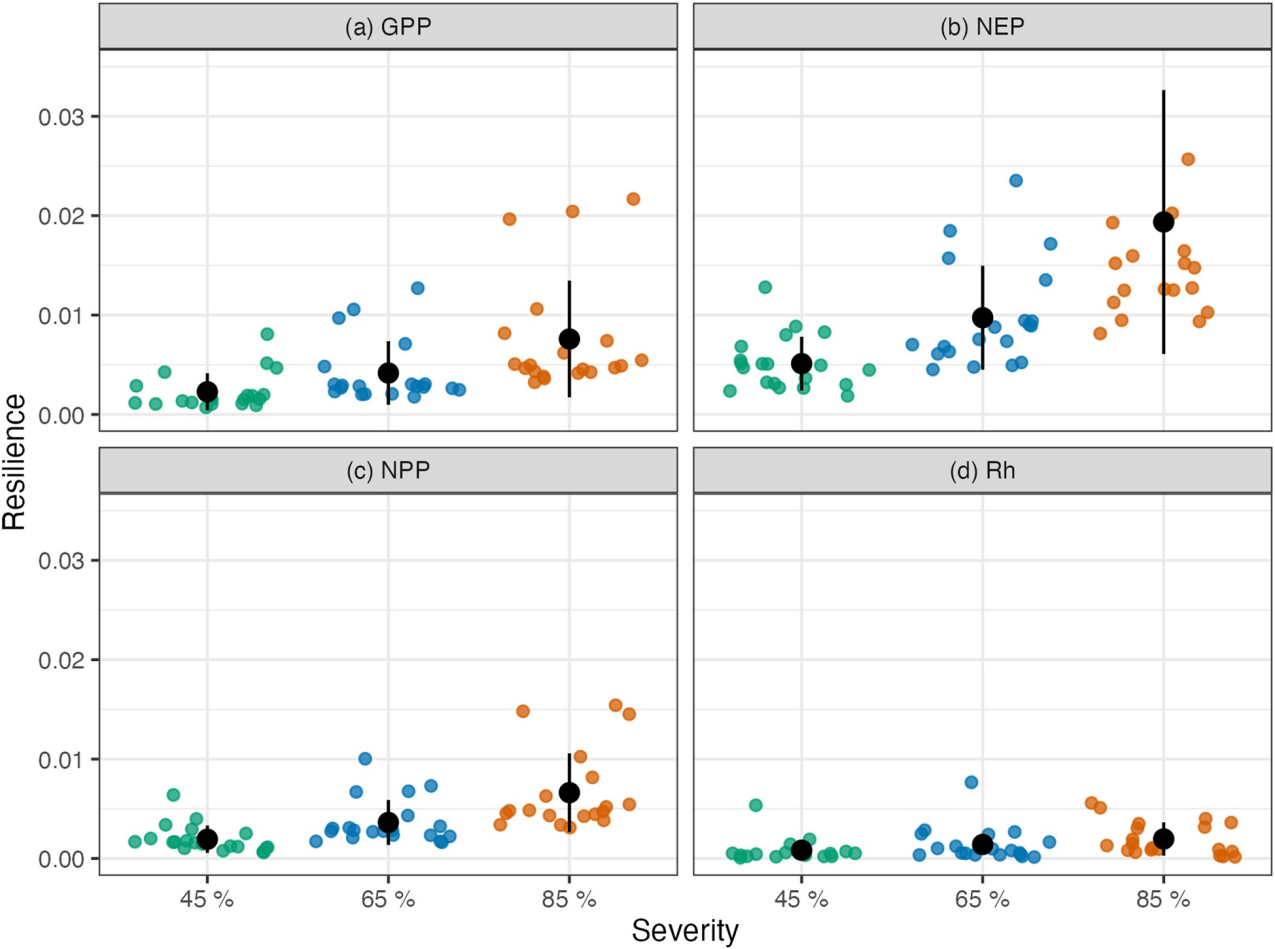
*Resilience*, the log‐ratio of disturbance to control flux, for gross primary production (a, GPP), net ecosystem production (b, NEP), net primary production (c, NPP), and heterotrophic respiration (d, Rh). Points are colored by disturbance severity. Each of the disturbance severity groups has 20 individual points, one for each of the idealized climate scenarios; the black point with error bar represents the mean *resilience* (Table 1) ± the standard deviation for each variable and treatment group. For clarity three *resilience* values greater than 0.035 are not shown here, but contributed to the mean and standard deviation.

**Figure 7:**
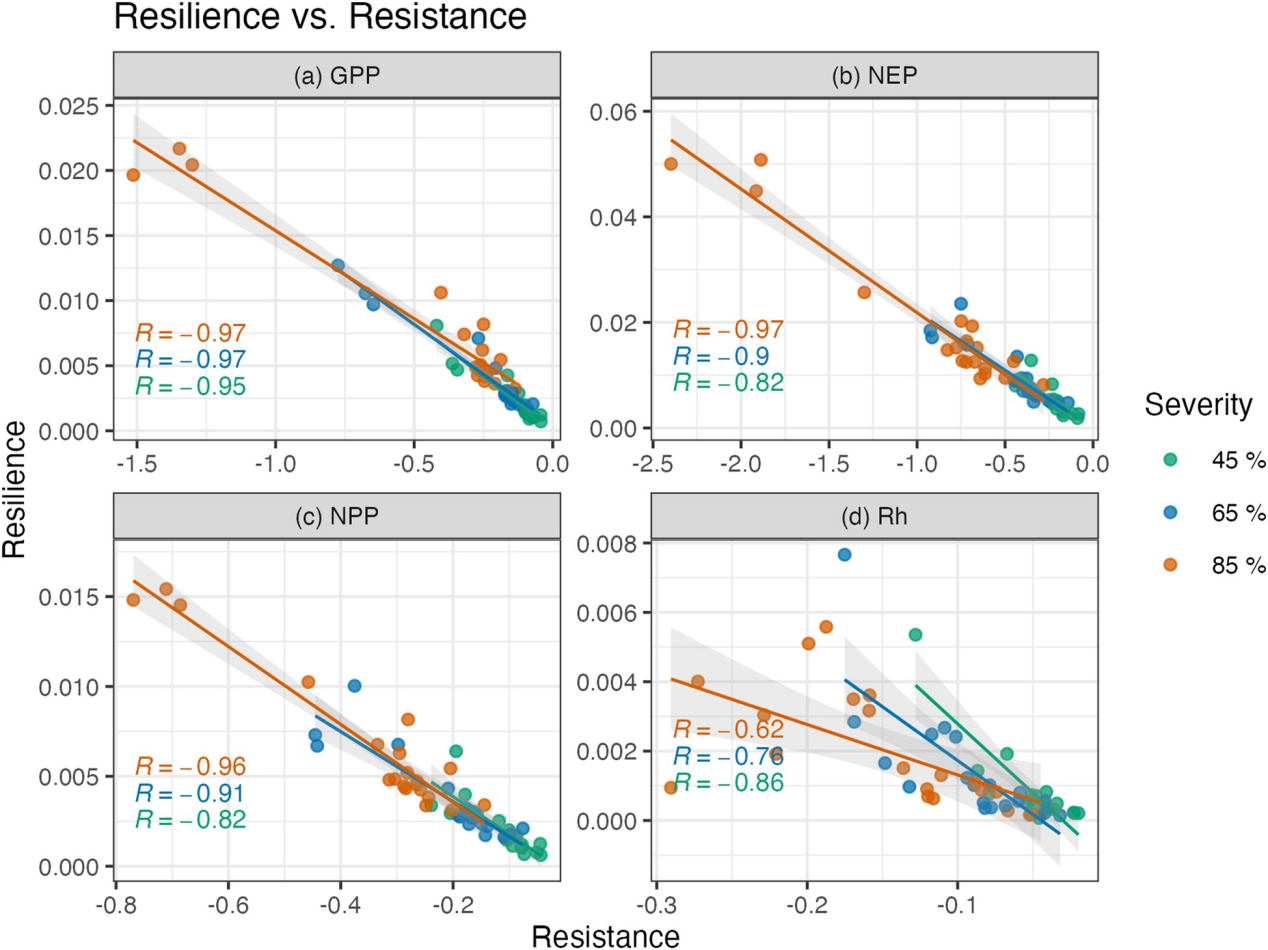
Scatter plot of *resistance* and *resilience* for gross primary production (a, GPP), net ecosystem production (b, NEP), net primary production (c, NPP), and heterotrophic respiration (d, Rh). Each of the FoRTE disturbance severity groups has 20 point lines, each one representing one of the 20 climate ensemble members. Trend lines show the linear regression with a 90% confidence interval.

Quantifying the flux response to disturbance in terms of resistance and resilience (Figures 5 and 6) allowed for the standardized comparison of treatment effects across different disturbance intensities and meteorological conditions. The mean resistance decreased with increasing treatment severity for all four C fluxes **(**Figure 5 and Table 1 **).** Mean GPP resistance decreased by 124% when the severity treatments increased from 45% to 65%, and by 142% from 65% to 85%. Similarly, the mean NEP resistance decreased by 145% and 189%, mean NPP resistance decreased by 120% and 134%, and mean Rh resistance decreased by 109% and 115% with increasing severity treatments. In contrast resilience roughly doubled with each increase in severity across variables (Figure 6 and Table 1). Ultimately ED exhibited an inverse linear relationship between resistance and resilience across the C fluxes and severity treatments (Figure 7).

**Table 1:** The Mean Resistance (Unitless) for Gross Primary Production (GPP), Net Ecosystem Production (NEP), Net Primary Production (NPP), and Heterotrophic Respiration (Rh) for Each of the FoRTE Disturbance Severity Groups (45%, 65%, and 85% Mortality)

Metric	Variable	45%	65%	85%
Resistance	GPP	−0.13	−0.23	−0.42
	NEP	−0.22	−0.45	−0.89
	NPP	−0.12	−0.20	−0.34
	Rh	−0.05	−0.09	−0.15
Resilience	GPP	0.002	0.004	0.008
	NEP	0.005	0.009	0.0019
	NPP	0.002	0.004	0.007
	Rh	0.0008	0.0014	0.002

*Note*. The lower part of the table shows mean resilience (year^−1^) for each variable and disturbance severity group.

**Figure 8:**
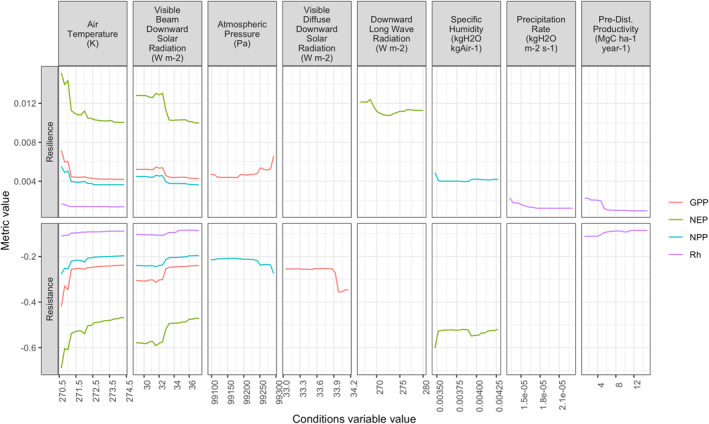
Partial dependence plots for resilience (top row) and resistance (bottom row) metrics, for most‐important variables from Random Forest analysis and four different model outputs (gross primary production, GPP; net ecosystem production, NEP; net primary production, NPP; and heterotrophic respiration, Rh). Each line provides a graphical depiction of the marginal effect of a meteorological variable on the output response, integrated over all other predictor values. Conditions variables include meteorological inputs (cf. Figure 2 and Figure S2 in Supporting Information S1) and the pre‐disturbance productivity (GPP in 2000 Figure S3 in Supporting Information S1).

As noted above, ambient conditions (i.e., which idealized climate scenario was used for each ED‐2.2 run) produced a wide spread in output fluxes, as well as high variability in *resistance* (Figure 5) and *resilience* (Figure 6). To explore which conditions are the strongest drivers of these changes, we used Random Forest models to rank the importance of conditions, meteorological inputs and pre‐disturbance productivity on *resistance* or *resilience* (Figure S4 in Supporting Information S1). GPP, NEP, NPP resistance results were most affected by air temperature, whereas Rh resistance was most affected by direct‐beam sunlight **(**Figure S5 in Supporting Information S1). Other meteorological variables that had relatively large (i.e., one of the three most important) effects on resistance (the top three most important variable rankings) include specific humidity, zonal wind, precipitation, and pre‐disturbance forest productivity. Rh was the C flux where pre‐disturbance forest productivity was one of the top five most important variables. Partial dependence plots visualize how each metric changed over the range of these most‐important inputs (Figure 8). Notably and consistently, warmer weather (air temperature) was associated with higher *resistance* (the model's outputs did not drop as far, on average) but lower *resilience* (it took longer for the C fluxes to return to pre‐disturbance levels). This pattern also held true for direct‐beam sunlight (visible beam downward solar radiation).

## 5 Discussion

Overall, the simulated UMBS forest C cycling disturbance responses and trends over time were consistent with observations and values reported in the literature. The NPP and NEP 2001 ensemble means are consistent with the published values of NPP and NEP UMBS observations from 1999 to 2003, which were 6.54 and 1.53 Mg C ha^−1^ respectively (Gough et al., 2008) (Figure 3). These ED‐2.2 outputs are also consistent with the mature UMBS simulated forests in Shiklomanov et al. (2020). In addition to generally being able to represent UMBS forests, our ED‐2.2 simulations demonstrated (a) a tradeoff between resistance and resilience (Figure 7), (b) the increasing lag (or attenuation) of disturbance effects as the C cycle proceeds from GPP to NPP to Rh (Figures 4, 5, 6), and (c) the importance of climate on forest C cycling response to mortality events.

The modeled relationship between resistance and resilience (Figure 7) is particularly interesting, as such a tradeoff has been observed experimentally but rarely in forests or in relation to C fluxes. Hillebrand et al. (2018) reported a significant correlation between functional resistance and resilience (with a Pearson's Square of *R* = −0.65, lower than the *R* = −0.9 observed in our simulations). However, our analysis is among the first to adapt the Hillebrand approach for analysis of C fluxes, with prior studies of resistance‐resilience trade‐offs focusing on microbial communities and biomass (Hillebrand et al., 2018). Demonstrating these dynamics thus constitutes a successful test for ED‐2.2; subjecting models to such relationship analyses (sensu, Collier et al., 2018) provides a more stringent constraint on, and test of, performance relative to, for example, assessing it against observed C stocks only (Keenan et al., 2013; Todd‐Brown et al., 2013).

Lagged impacts, a noticeable feature in Figures 4, 5, 6, are an important aspect of how terrestrial ecosystems and the C cycle respond to many types of disturbance (Bloom et al., 2020; Frank et al., 2015). For example, GPP responds rapidly to changes in environmental conditions, while NPP recorded in tree rings typically lags climate changes (Babst et al., 2014) due to plants’ use of nonstructural carbohydrates synthesized in previous months to years (Richardson et al., 2013). In contrast, respiration can take the better part of a decade to recover after insect‐driven mortality (Moore et al., 2013) or canopy browning (Bond‐Lamberty et al., 2012). The muted response and lagged behavior of Rh seen here is consistent with much empirical work. One possible explanation is that soil respiration is driven by heterotrophic and autotrophic sources that are buffered, at least to some degree, from abrupt changes in aboveground conditions (Ruehr et al., 2010); utilize C of a wide range of ages (Trumbore, 2000); and may be only loosely coupled with aboveground fluxes (Bader et al., 2016; Baldocchi et al., 2006).

The strong effects of meteorological drivers on resistance and resilience suggest that forest response to mortality events may be highly contingent on climatic conditions leading up to, during, and immediately after disturbance. On one hand, this is expected: climate affects plant photosynthesis and growth (Anav et al., 2015; Charney et al., 2016) and most other aspects of the C cycle, including recovery from disturbance (Anderson‐Teixeira et al., 2013). Whether more‐productive forests will be more resistant and/or resilient to disturbance is unclear (Campos et al., 2013; Stone et al., 1996; Yi & Jackson, 2021) but has meaningful consequences. Rates of forest recovery (which should be tightly tied to the resilience metrics used here) generally increase with CO_2_, temperature, and water availability (Anderson‐Teixeira et al., 2013; Goetz et al., 2006). Among the ED‐2.2 meteorological drivers, air temperature and direct‐beam sunlight were the most consistent drivers affecting forest disturbance response (Figure 8): in general higher values were associated with higher resistance (less impact) but lower resilience (slower recovery). This was true for three of the four major C fluxes (GPP, NPP, and NEP); heterotrophic respiration (Rh) metrics were more affected by precipitation and pre‐disturbance productivity. Moreover, an important caveat here is that our experimental design cannot easily disentangle if the condition (in particular, productivity) of the forest entering the FoRTE disturbance event was more important than the post‐event climate; in other words, does forest response depend more on pre‐disturbance or post‐disturbance conditions?

A number of additional limitations in our experimental design and model should be noted. The girdling treatment (Högberg et al., 2001) used in the FoRTE and preceding FASET experiments produces effects closest, mechanistically, to those of phloem‐boring insects and pathogens (Dietze & Matthes, 2014). Although ED‐2.2 does include a representation of mortality due to C starvation, there is currently no way to introduce an exogenous disturbance to this scheme that is analogous to a girdling treatment (Longo et al., 2019). More generally, the wide variety of biotic and abiotic ecosystem disturbances are poorly represented in ecosystem models (Hicke et al., 2011). The harvest—aboveground biomass removal—mechanism we used in this study should approximate the years immediately after girdling, when photosynthate supply to the roots is terminated but widespread mortality and in particular aboveground decay have not yet occurred (Högberg et al., 2001). The post‐disturbance modeling results might increasingly diverge from the real world over time, however, as the “missing” stem biomass (removed from the site in the model, but not in the real world) falls to the ground and decays, releasing CO_2_ and librerating nutrients and thereby affecting Rh and NEP (Harmon et al., 2011). We suggest that implementing a C starvation disturbance type in ED‐2.2, and/or other prominent ecosystem‐scale models, should be a high priority in the future, following the example of for example, Frasson et al. (2015).

Additionally, our approach of using single‐year meteorology repeated over the course of the runs ignores, by design, year‐to‐year variability of weather and the impacts that variability may have on the ecosystem. Lacking these dynamics, we are unable to determine if or to what extent the effects of weather variability have on the forest disturbance response which may have contrasting effects on productivity and ecosystem vulnerability (Holmgren et al., 2013), or (as noted above) if the pre or post‐disturbance climate has stronger implications for the ecosystems’ recovery. Such interannual variability can significantly affect ecosystem to regional carbon fluxes, both in this upper Midwest forest region (Desai, 2010) and more generally Shiga et al. (2018). However our approach is fundamentally a strength given that one of our objectives was to investigate interactions between climate and disturbance severity, which was possible given our experimental design. Furthermore, since ecophysiological models such as ED‐2.2 are sensitive to meteorological inputs, these conditions themselves may lead to mortality events as seen in Figure 3, which emphasizes the need for working with an ensemble of results as opposed to a single time series of model inputs and outputs.

These results have potential real‐world implications for both forest managers and future analyses of how temperature of deciduous forests may respond to changing disturbance regimes. Our findings suggest that warmer and wetter conditions are associated with forests that are more vulnerable to a mortality event, but also may recover more quickly. Forest managers have no control over future climate conditions of the upper Midwest, but may be able to use the trade‐off between resistance and resilience and their relationship with forecasted climate to their advantage. A central goal of forest management is achieving desired and predictable structural and functional conditions (Corace et al., 2012), and in particular canopy complexity (Crow et al., 2002), which is closely linked with resilience (Stuart‐Haëntjens et al., 2015). Analyses such as ours may also help managers mitigate “resilience debt” (Johnstone et al., 2016), in which accumulating stresses render an ecosystem less resistant or resilient.

Our results have implications for the ongoing FoRTE experiment as well, and for future investigations into forest disturbance response. Predicting the different possible timescales of C flux response has implications for sampling frequency and priority; for example, post‐disturbance Rh observations may need to be collected over a relatively extended period of time in order to see the full extent of disturbance response. Model parameter sensitivities can also inform field sampling priorities (Shiklomanov et al., 2020). Such a feedback loop between sampling and modeling thus benefits both areas, and builds on recent calls to improve model‐experimental communication and productivity (Dietze et al., 2013; Medlyn et al., 2015). In addition, the field validation of a strong C cycling resistance and resilience tradeoff would provide powerful and novel evidence that long‐term C cycling responses (i.e., resilience) can be forecasted from initial changes (i.e., resistance).

In conclusion, our findings highlight the variability of C flux response and recovery to a disturbance within a given ecosystem, and that this variability is in part related to climatic conditions. This comparative synthesis across C flux variables, modeling results, and observations was strengthened by using standardized and normalized metrics to quantify multiple dimensions of ecosystem stability. However, more work is needed to better understand the interactions and consequences of interactions between climate, disturbances, and ecosystem stability. Future work could evaluate the importance of pre‐disturbance productivity versus post‐disturbance weather by analyzing forest recovery from disturbances captured by Landsat, for example (Kennedy et al., 2010; Williams et al., 2014) or the effects of interannual climate variability. Improving the representation of disturbances within ecosystem models is fundamental to advancing model based studies. Lastly, continued analysis of the open‐science (Atkins et al., 2020) observations collected from the FoRTE UMBS experiment will improve our understanding of the present and future trajectories of temperate deciduous forests in a changing climate.

## Supporting information

Supporting Information S1

## Data Availability

ED‐2.2 was used to conduct the model simulations, it is openly developed at https://github.com/edmodel/ed2. Inputs, final results, workflows, and code used to visualize manuscript results are available at https://github.com/forTExperiment/forte-disturbance.
