# Supplementary material for: Climate Drives Modeled Forest Carbon Cycling Resistance and Resilience in the Upper Great Lakes Region, USA
Source: J Geophys Res Biogeosci. 2022 Jan 13;127(1):e2021JG006587. doi: 10.1029/2021JG006587 (PMC9287023; doi:10.1029/2021JG006587)
Supplement: Supplementary file 1 — Supporting Information S1 [file JGRG-127-0-s001.docx]

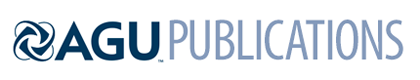


*Journal of Geophysical Research*

Supporting Information for

**Climate drives modeled forest carbon cycling resistance and resilience in the Upper Great Lakes Region, USA**

**Kalyn Dorheim^1^, Christopher M. Gough^2^, Lisa Haber^2^, Kayla C. Mathes^2^, Alexey Shiklomanov^3^, Ben Bond-Lamberty^1^**

^1^ Joint Global Change Research Institute, Pacific Northwest National Laboratory, College Park, MD 20740 USA

^2^Department of Biology, Virginia Commonwealth University, Richmond, VA 23284 USA

^3^NASA Goddard Space Flight Center, Greenbelt, MD, 20771 USA

**Contents of this file**

Figures S1 to S5


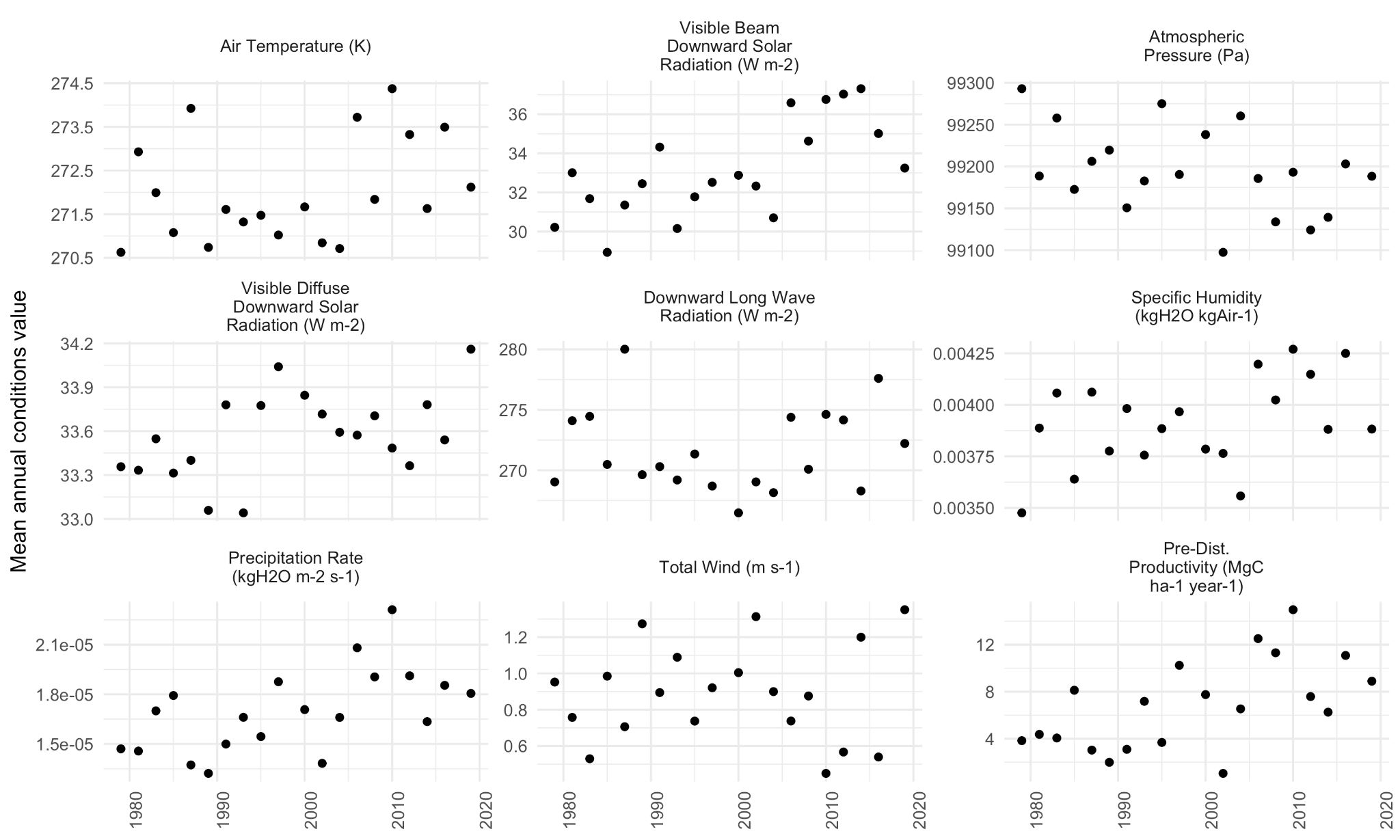


Figure S1. Mean annual meteorological values sampled from the site historical record. Each mean annual value corresponds to the monthly averages in Figure 2 and are repeated from 1900 to 2050 to form the idealized climate scenarios for this study. The pre-disturbance productivity refers to the GPP in 2000.


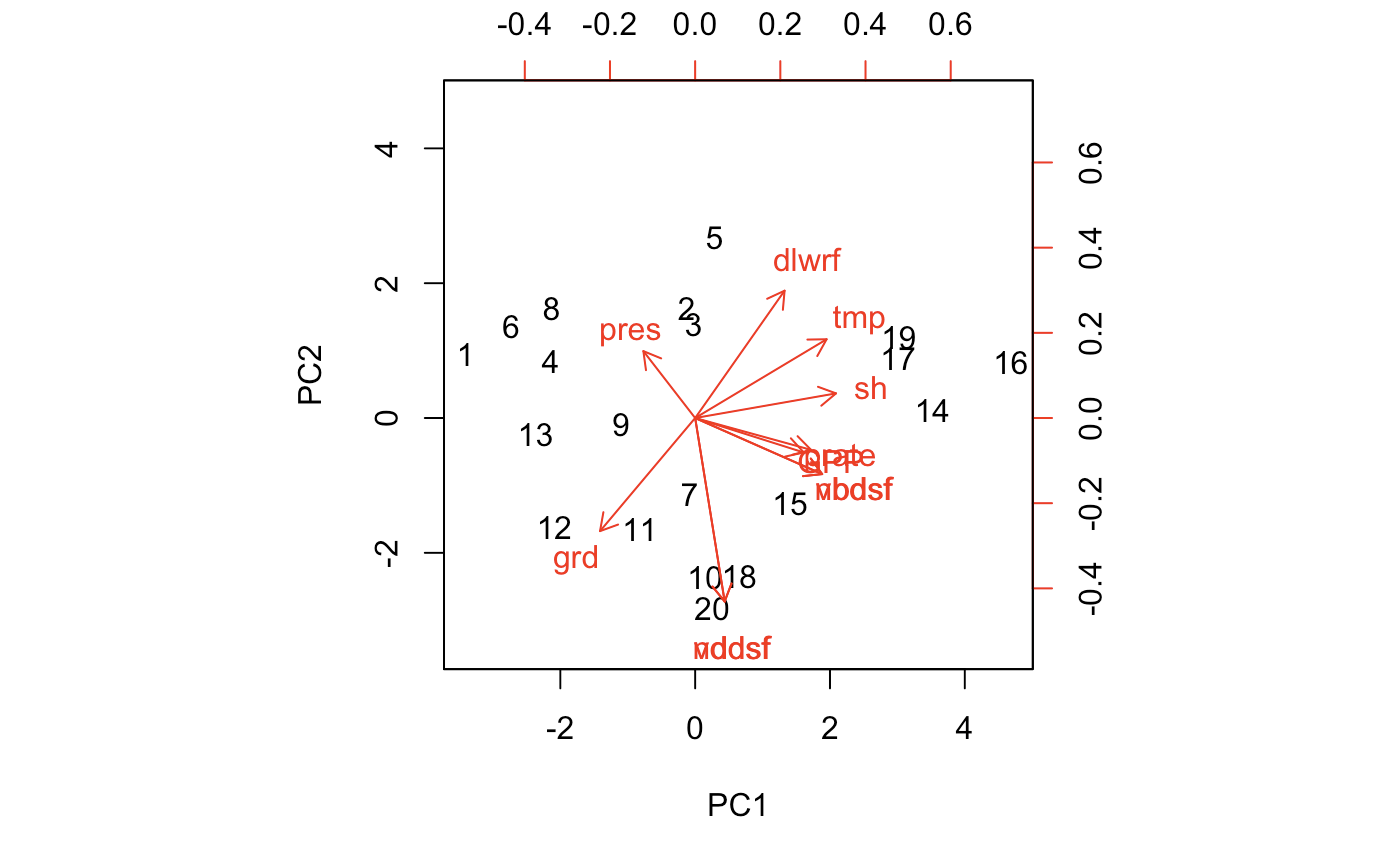


Figure S2. Principal Coordinate (PC) plot for the meteorological data. The *vbdsf* variable (visible beam downward solar radiation) is highly correlated with *nbdsf* (near infrared beam downward solar radiation), such that their arrows and titles overlap each other; *vddsf* (visible diffuse) and *nddsf* (near infrared diffuse) are similarly correlated and overlapping.


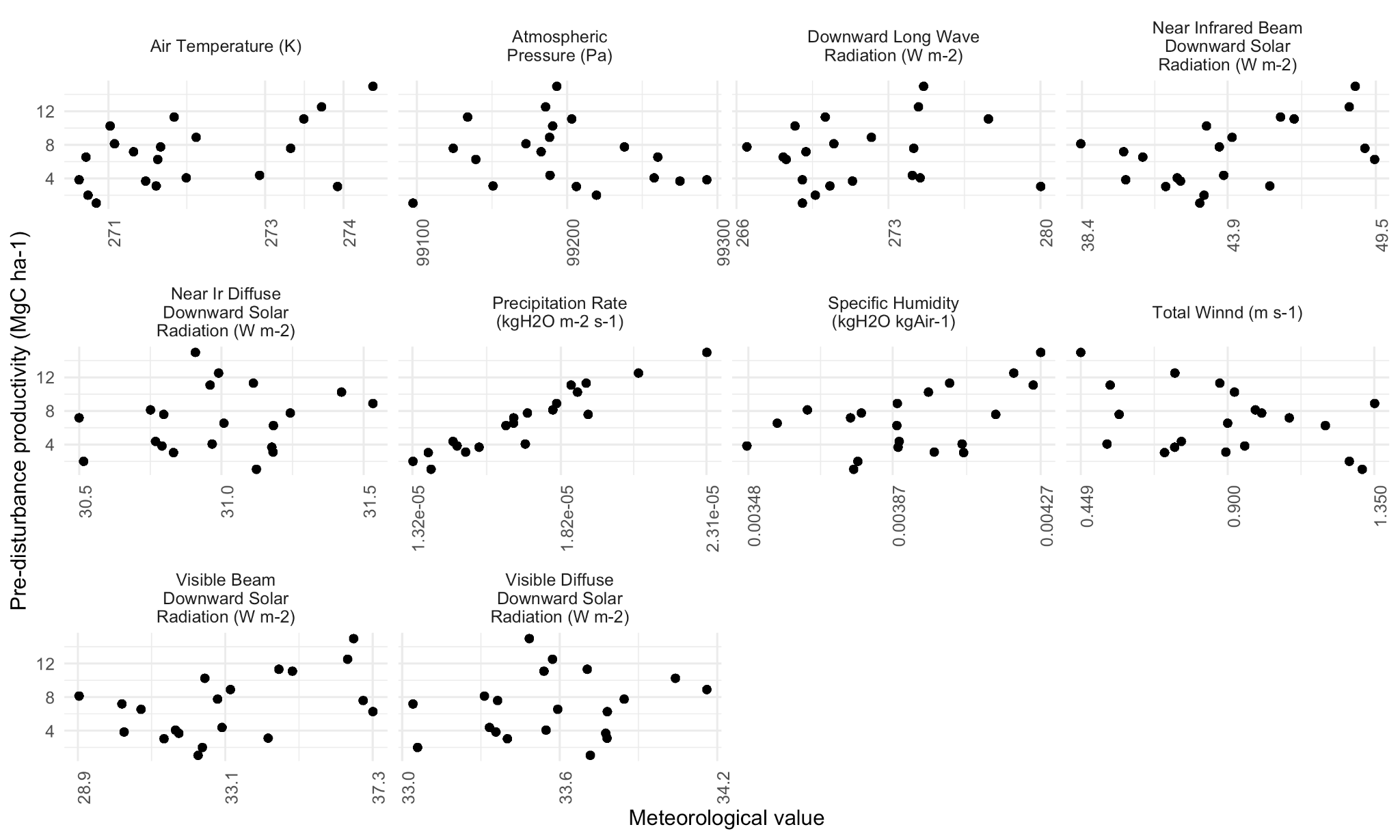


Figure S3. Mean annual meteorological values versus pre-disturbance productivity (i.e. gross primary production) in 2000. Each panel shows a different meteorological variable.


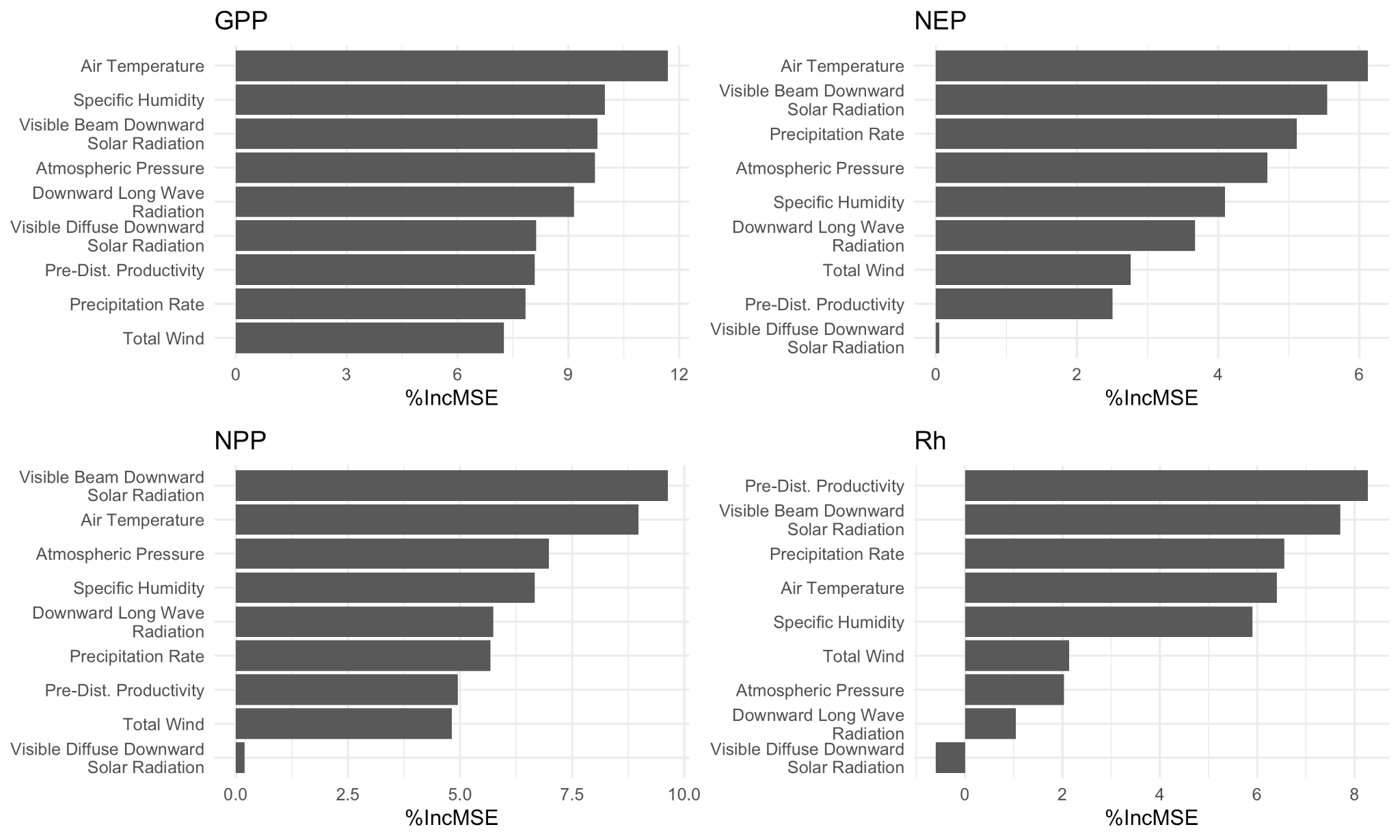


Figure S4. Variable importance plots for *resistance*, calculated from Random Forest model (see Methods), for the four main carbon flux outputs: gross primary production (GPP), net primary production (NPP), net ecosystem production (NEP), and heterotrophic respiration (Rh).


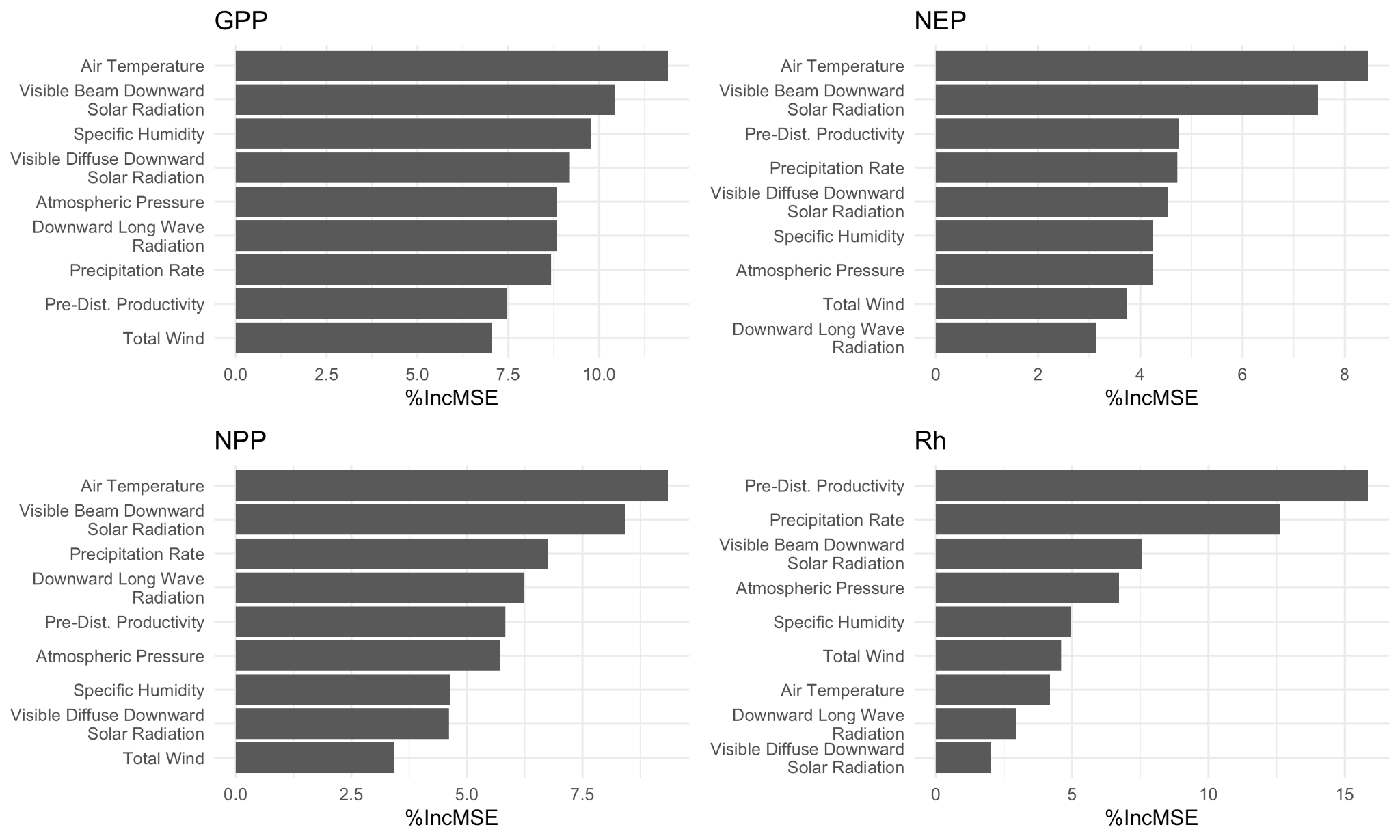


Figure S5. Variable importance plots for *resilience*, calculated from the Random Forest model (see Methods). Panels are as in Figure S4.
